# Supplementary material for: Hypothalamic Rax+ tanycytes contribute to tissue repair and tumorigenesis upon oncogene activation in mice
Source: Nat Commun. 2021 Apr 16;12:2288. doi: 10.1038/s41467-021-22640-z (PMC8052410; doi:10.1038/s41467-021-22640-z)
Supplement: Supplementary file 1 — Supplementary Information [file 41467_2021_22640_MOESM1_ESM.pdf]

Supplementary Information for

Hypothalamic Rax$^+$ tanycytes contribute to tissue repair and tumorigenesis upon oncogene activation in mice

Authors: Wenhui Mu[1]†, Si Li[1,2]†, Jingkai Xu[1,3]†, Xize Guo[1,2], Haoda Wu[1,2], Zhenhua Chen[1,2], Lianyong Qiao[1], Gisela Helfer[4], Falong Lu[1,2], Chong Liu[5], Qing-Feng Wu[1,2,6,7]*

This PDF file includes:

Supplementary Figures and Legends

Supplementary Figures 1-10

Supplementary Data and Tables

Supplementary Data 1-3

Supplementary Tables 1-2

Supplementary Figure 1

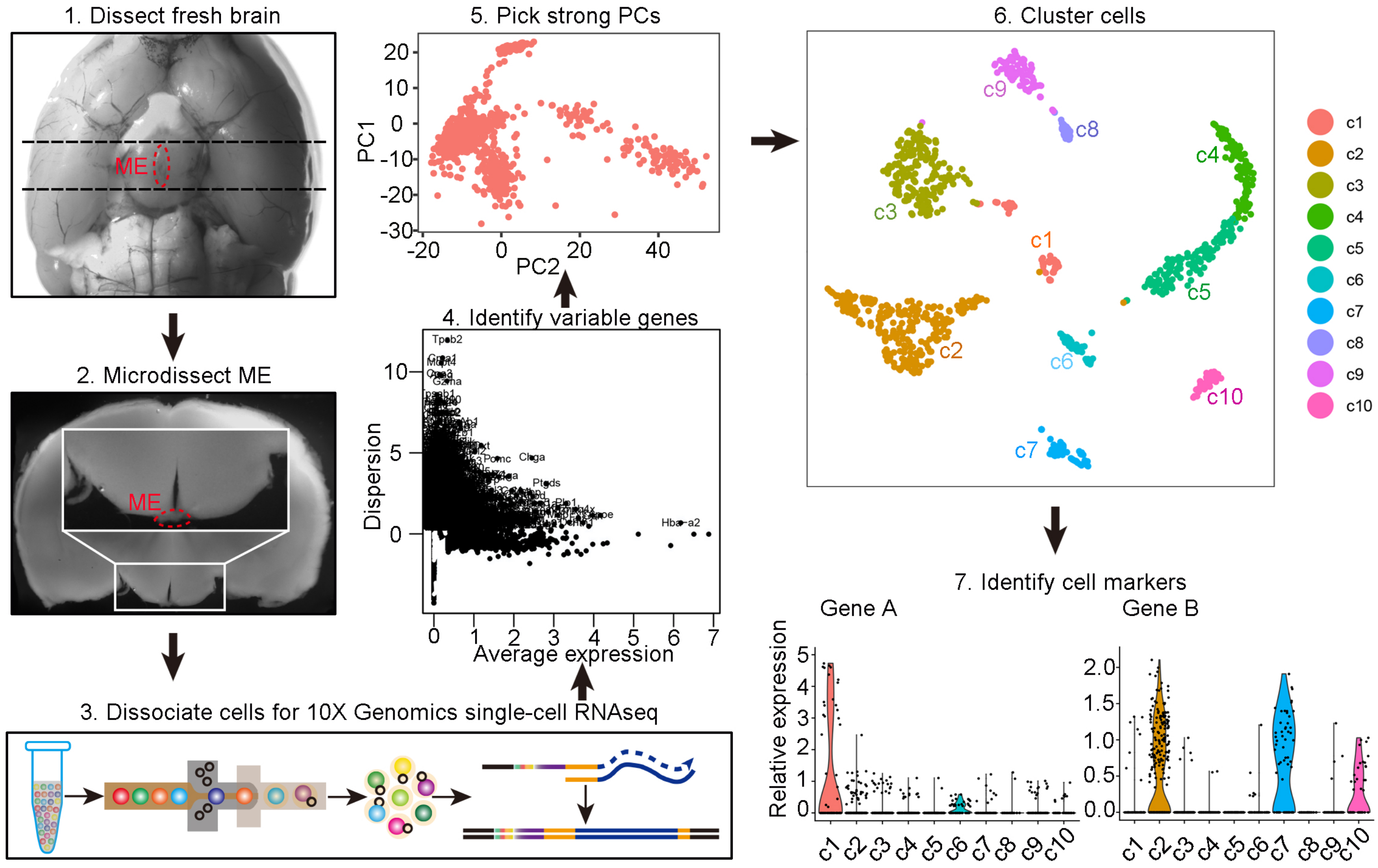

Supplementary Figure 1. Single-cell RNA sequencing of cells in the median eminence (ME). Schematic diagram of single-cell transcriptomic analysis. ME tissues were collected from 10 adult mice and pooled together for digestion and acute dissociation. The single-cell cDNA libraries were generated for single-cell RNA sequencing using 10×Genomics (Chromium) platform. After applying cell filtering algorithm to remove unqualified cells, we then identified variable genes, performed principal component (PC) analysis and dimensionality reduction with spectral *t*-distributed stochastic neighbor embedding (tSNE) to cluster cells.

Supplementary Figure 2

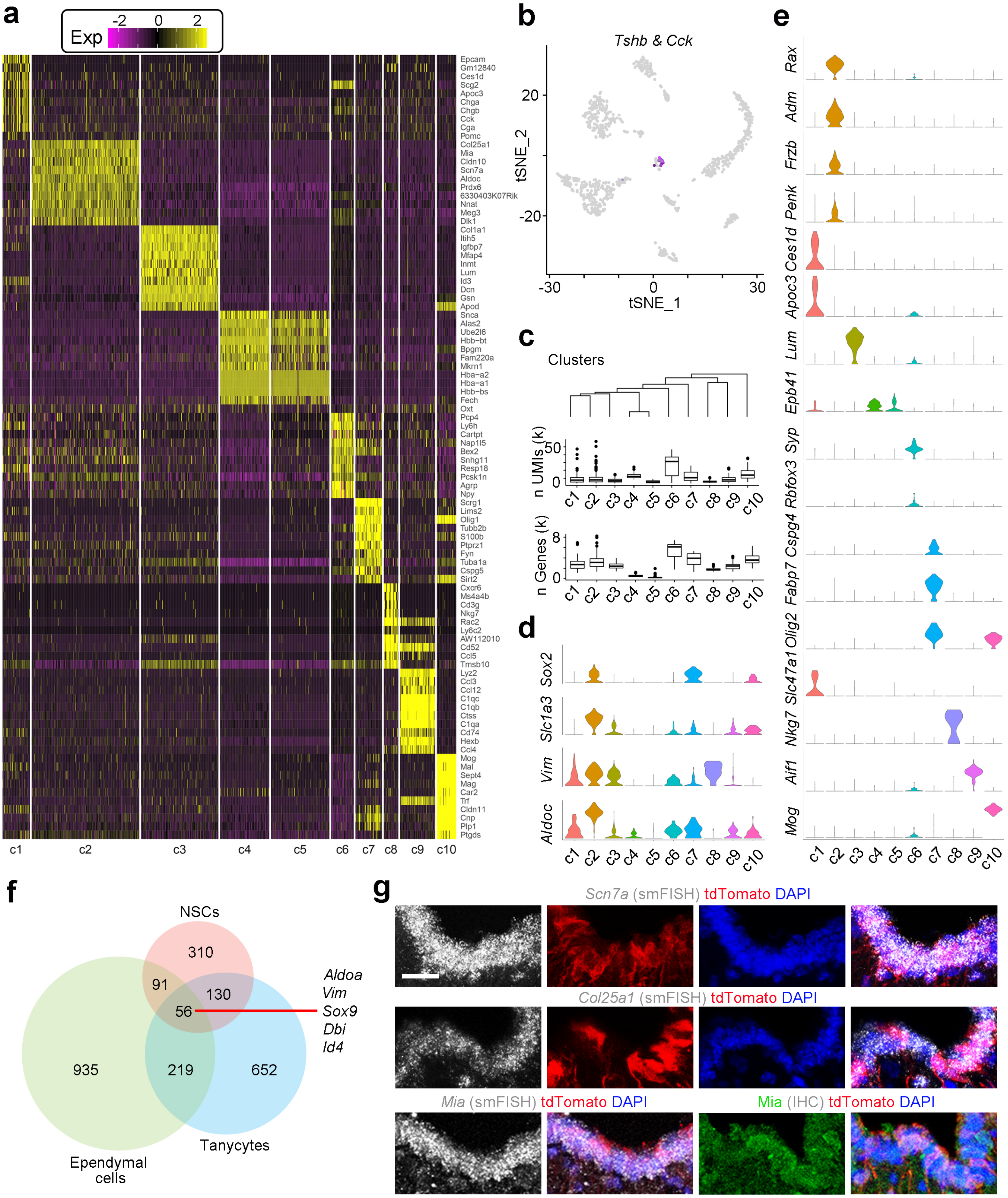

Supplementary Figure 2. Single-cell RNAseq analysis of cells in ME. a, Heat map of top 10 marker genes for each cluster. **b**, tSNE scatter plot showing the specific expression of *Tshb* and *Cck* in pars tuberalis cells (c1 cluster). Exp, expression. **c**, Shown are cell cluster dendrogram, number of unique molecular identifiers (UMIs) per cluster and genes detected per cluster. Boxes represent interquartile range (IQR) and whiskers extend to ±1.5 IQR (n=33, 243, 175, 111, 132, 62, 79, 44, 51 and 60 for c1, c2, c3, c4, c5, c6, c7, c8, c9 and c10, respectively). **d-e**, Shown are violin plots exhibiting expression of cell-type marker genes. **f**, Venn diagram showing the shared molecular signatures among NSCs, ependymal cells and tanycytes. A published database related to NSCs and epenedymal cells is used for comparison [1]. **g,** Representative images of traced tdTomato^{+} cells stained for specific tanycyte markers including *Scn7a*, *Col25a1* and *Mia* in Rax-CreER^{T2}::Ai14 mice. The animals were induced with tamoxifen and sacrificed for smFISH analysis or staining at 1 day post induction (dpi). Scale bar, 20 µm.

Supplementary Figure 3

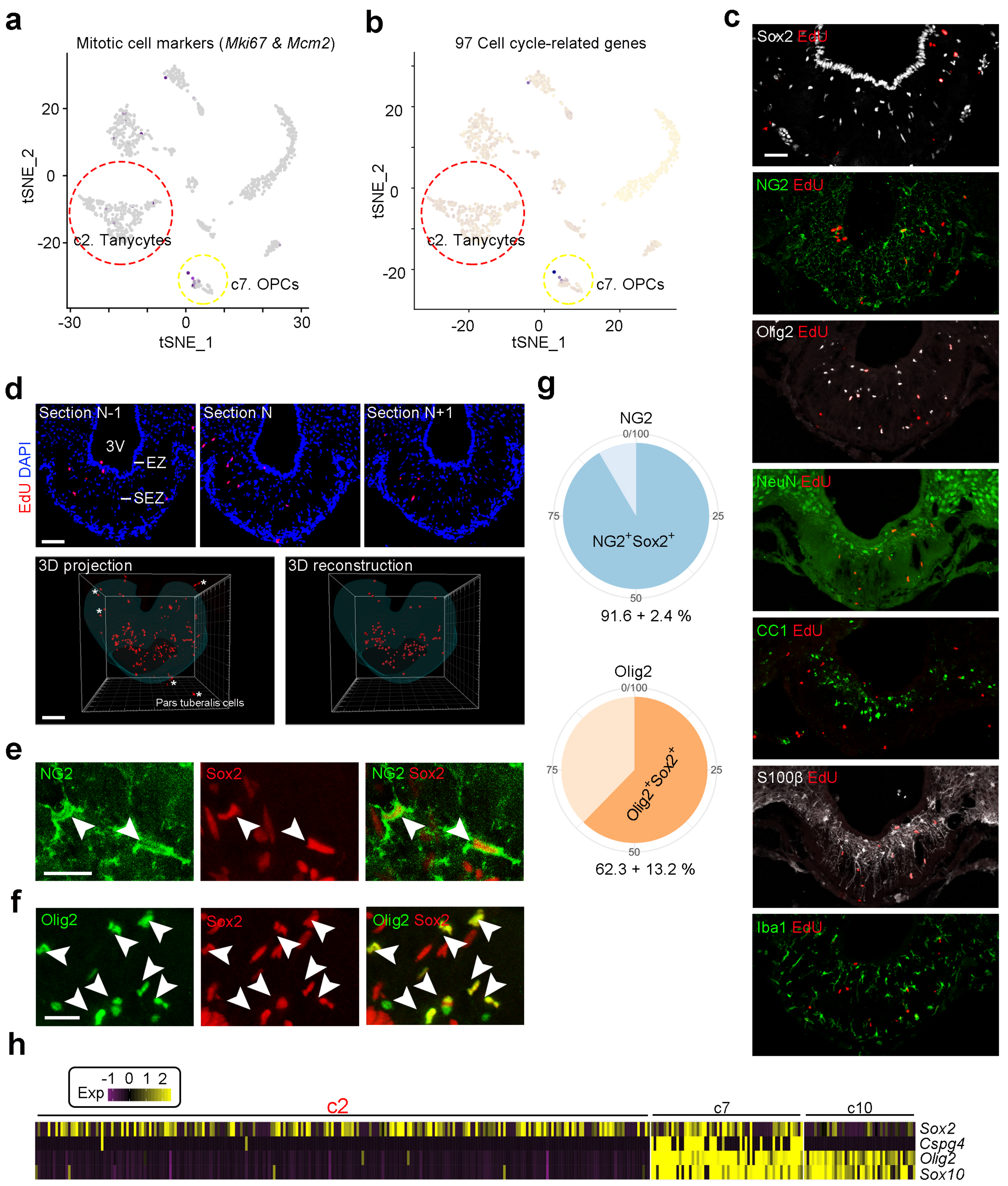

Supplementary Figure 3. Cell-type characterization of mitotic cells in ME. a, tSNE feature plot showing the expression of *Mki67* and *Mcm2* in different cell clusters. **b,** Cell cycle-related gene set including 43 S-phase genes and 54 G2/M-phase genes are relatively enriched in oligodendrocyte precursor cells (OPCs) rather than tanycytes. **c**, Sample confocal images showing the colabeling of EdU with different cell type markers Sox2, NG2, Olig2, NeuN, CC1, S100β and Iba1. Scale bar, 50 µm. **d**, Serial coronal brain sections showing the chase of mitotic cells at 1 day post a single pulse with EdU in ME. Three-dimensional (3D) projection and reconstruction of the hypothalamic proliferative niche provide a global view of dividing cell distribution. Scale bars, 50 µm (top) and 100 µm (bottom). **e-g**, Colocalization of OPC markers NG2 and Olig2 with Sox2. Shown are representative images stained for NG2, Olig2 and Sox2 (e and f) and pie charts quantifying the percentage of double labeled cells among $NG2^+$ or $Olig2^+$ cells (g). Arrowheads indicate the double labeled cells. Scale bars, 20 µm. **h,** Heat map showing the relative expression of *Sox2*, *Cspg4* (encoding NG2), *Olig2* and *Sox10* in c2, c7 and c10 cell clusters. Exp, expression.

Supplementary Figure 4

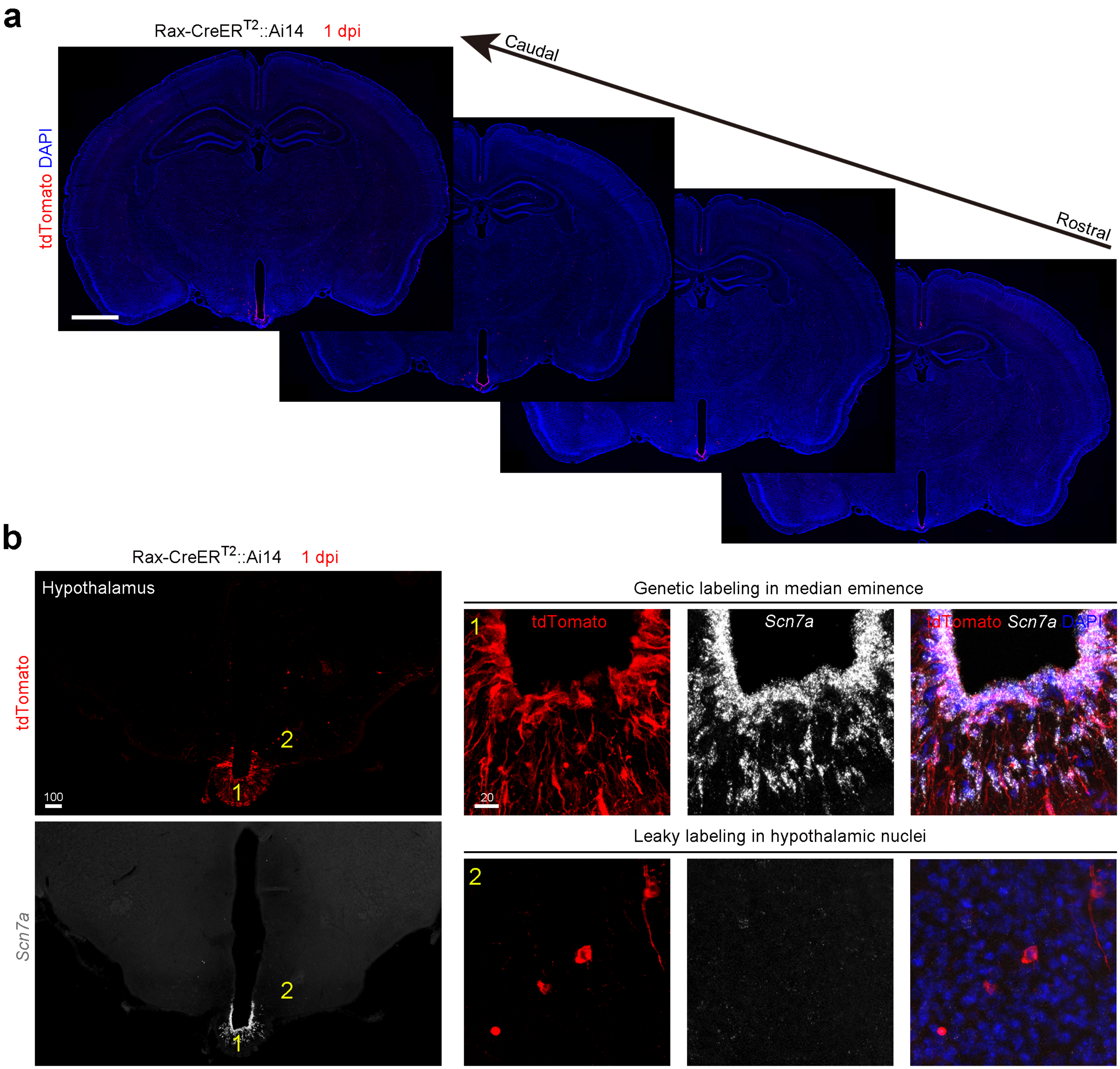

Supplementary Figure 4. Lineage tracing of tanycytes using Rax-CreER^{T2} knockin mouse line. **a**, Representative images showing the genetic labeling of tanycytes lining the third ventricle using Rax-CreER^{T2}::Ai14 mouse line at 1 day post induction (dpi) with tamoxifen. The arrow signifies the order of coronal brain sections along rostrocaudal axis. Scale bar, 1 mm. **b**, Representative images of traced tdTomato^{+} cells stained for tanycyte marker *Scn7a* in Rax-CreER^{T2}::Ai14 mice at 1 day post tamoxifen induction. The results show the precise genetic labeling of tanycytes in ME and sparse leaky labeling of tdTomato^{+} cells in hypothalamic nuclei. Scale bar, 50 μm (left) and 10 μm (right).

Supplementary Figure 5

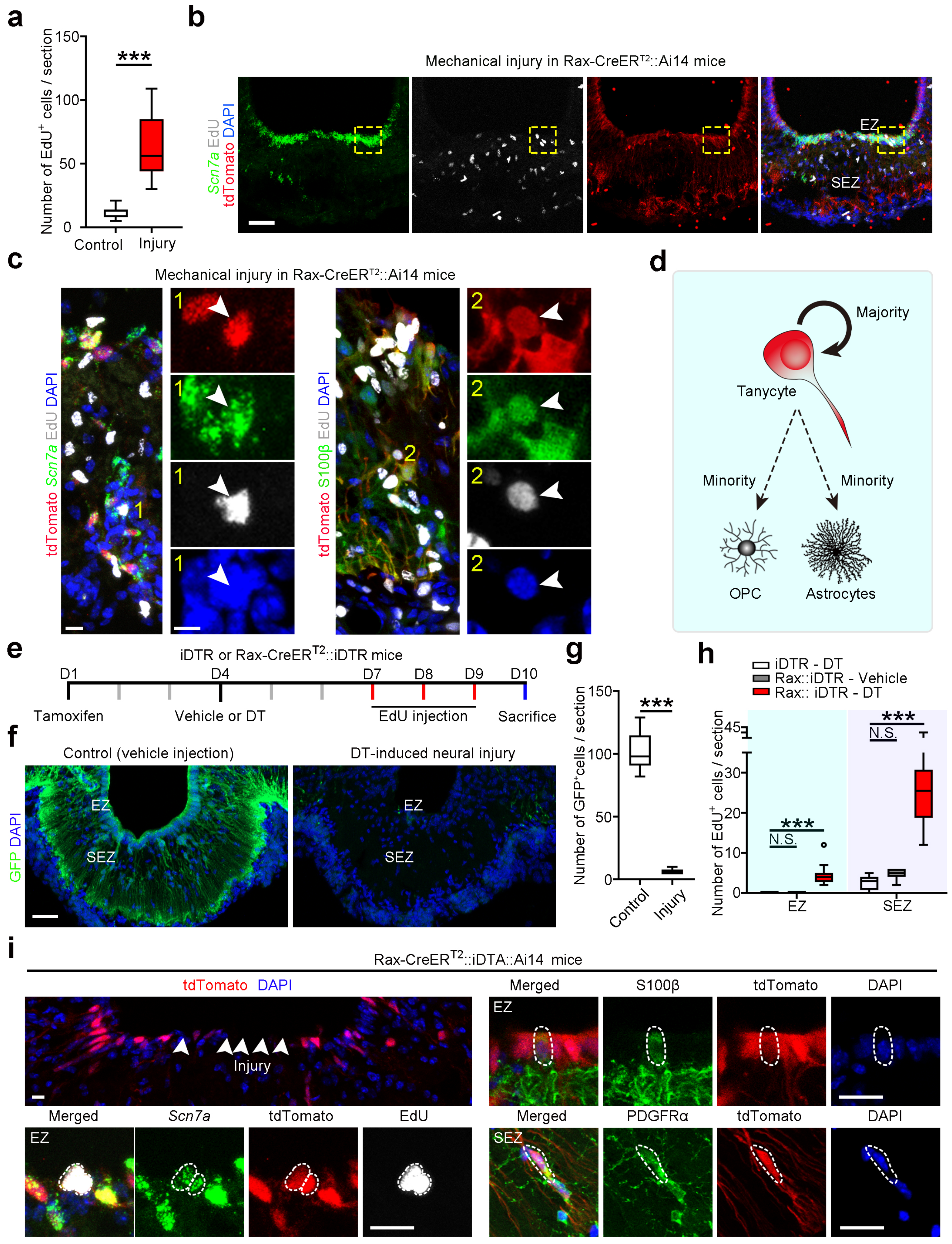

Supplementary Figure 5. Activation of Rax^+ tanycytes by neural injury. **a**, Quantification of the total dividing cell number in control and injured mice, indicating that mechanical injury enhances mitotic cell division in ME. Data are presented as box plot. Boxes represent IQR, whiskers extend to ±1.5 IQR and significance was analyzed by unpaired two-tailed Student's *t* test (n=11 and 10 sections from 3 animals for control and injury groups). ***, $p < 0.001$. **b**, Sample confocal images showing the triple labeling of tdTomato, EdU and *Scn7a* in Rax-CreER^{T2}::Ai14 mice receiving mechanical injury. Boxed images are shown in Fig. 3e. Scale bar, 50 µm. **c**, Sample confocal images showing that mechanical injury induces the self-renewal of tanycytes (tdTomato$^+$EdU$^+$*Scn7a*$^+$) and their differentiation into astrocytes (tdTomato$^+$EdU$^+$S100β$^+$). The tanycytes in ME were labeled by a single injection of tamoxifen to adult Rax-CreER^{T2}::Ai14 mice, followed by mechanical injury of ME at 7 dpi. The animals received three daily EdU injection at 1 day after injury and were then sacrificed for in situ hybridization with probe against *Scn7a* or immunostaining with antibodies against S100β or Olig2. Scale bars, 10 µm (left) and 5 µm (right). **d**, A model showing that ME tanycytes predominantly undergo self-renewal upon injury and a minority of them display the potential to differentiate or transdifferentiate into OPCs and astrocytes. **e**, Experimental paradigm for targeted neural injury and examination of damage repair following injury. **f**, Sample confocal images of ME show the genetic ablation of EGFP$^+$ tanycytes after diphtheria toxin (DT) administration. Scale bar, 50 µm. **g**, Quantification of the number of GFP$^+$ cells before and after DT injection. Data are presented as box plots and *p* value was analyzed by unpaired two-tailed Student's *t* test (n=12 and 10 sections from 3 animals for control and injury groups). **h**, Quantification of the dividing cell number in Fig. 3f. Boxes represent interquartile range (IQR), whiskers extend to ±1.5 IQR and *p* values were computed by one-way ANOVA with Sidak's multiple comparison test (n=22, 12, 18, 22, 12 and 18 sections from at least 3 mice from

left to right boxes). ***, $p < 0.001$. **i**, Representative confocal images showing the genetic ablation of partial tanycytes in ME and the costaining of tdTomato with *Scn7a*, S100β or PDGFRα in a subpopulation of tanycyte-derived cells within Rax-CreER^{T2}::iDTA::Ai14 mice subjected to genetically-induced injury. The animals were given three daily injections of tamoxifen to induce neural injury. Scale bars, 10 µm. Source data are provided as a Source Data file. The precise *p* values are summarized in Supplementary Data 3.

Supplementary Figure 6

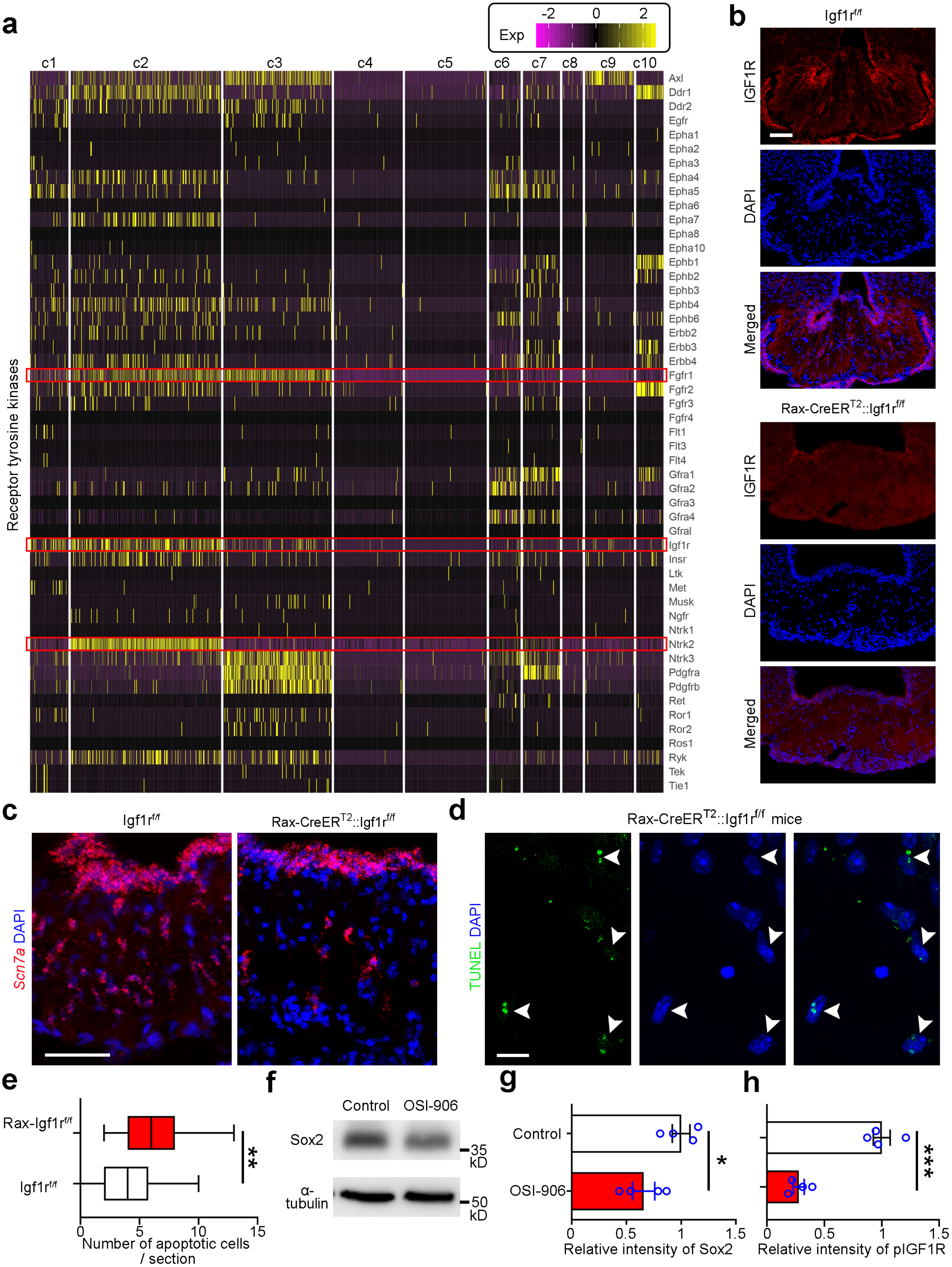

Supplementary Figure 6. Igf1r signaling is involved in maintaining tanycytes. a, Systematic analysis of the expression of 51 receptor tyrosine kinases (RTKs) in different cell clusters. *Igf1r*, *Fgf1r* and *Ntrk2* are highlighted in red. Exp, expression. **b**, Representative confocal images showing the expression of Igf1r in the ME of control mice and its genetic deletion in Rax-CreER^{T2}::Igf1r$^{f/f}$ mice receiving five daily tamoxifen injection. Scale bar, 50 µm. **c**, Representative confocal images showing the reduction of *Scn7a*$^{+}$ tanycytes in Igf1r-deficient mice as compared with control animals. The *Scn7a* mRNA in tanycytes was detected by smFISH. Scale bar, 50 µm. **d**, Sample confocal images indicating the abundant TUNEL signal in the ME of Rax-CreER^{T2}::Igf1r$^{f/f}$ mice at 1 month post injection (mpi) with tamoxifen. Scale bar, 10 µm. **e**, Quantification of the apoptotic cell number in control and Igf1r-deficient mice. Data are presented as box plot wherein boxes represent IQR and whiskers extend to ±1.5 IQR and significance was analyzed by unpaired two-tailed Student's *t* test (n=24 sections from 5 mice for Igf1r$^{f/f}$ group and 19 sections from 4 mice for Rax-Igf1r$^{f/f}$ group). ** $p < 0.01$. **f**, Immunoblotting analysis of Sox2 and α-tubulin expression in cultured neural stem cells treated with 1 µM OSI-96. **g-h**, Densitometric quantification of normalized Sox2 (g) and pIGF1R (h) expression detected by Western blot. Values represent mean ± SEM (n=4 biologically independent samples). *P* values were analyzed using unpaired two-tailed Student's *t* test. *, $p < 0.05$, ***, $p < 0.001$. Source data are provided as a Source Data file. The precise *p* values are summarized in Supplementary Data 3.

Supplementary Figure 7

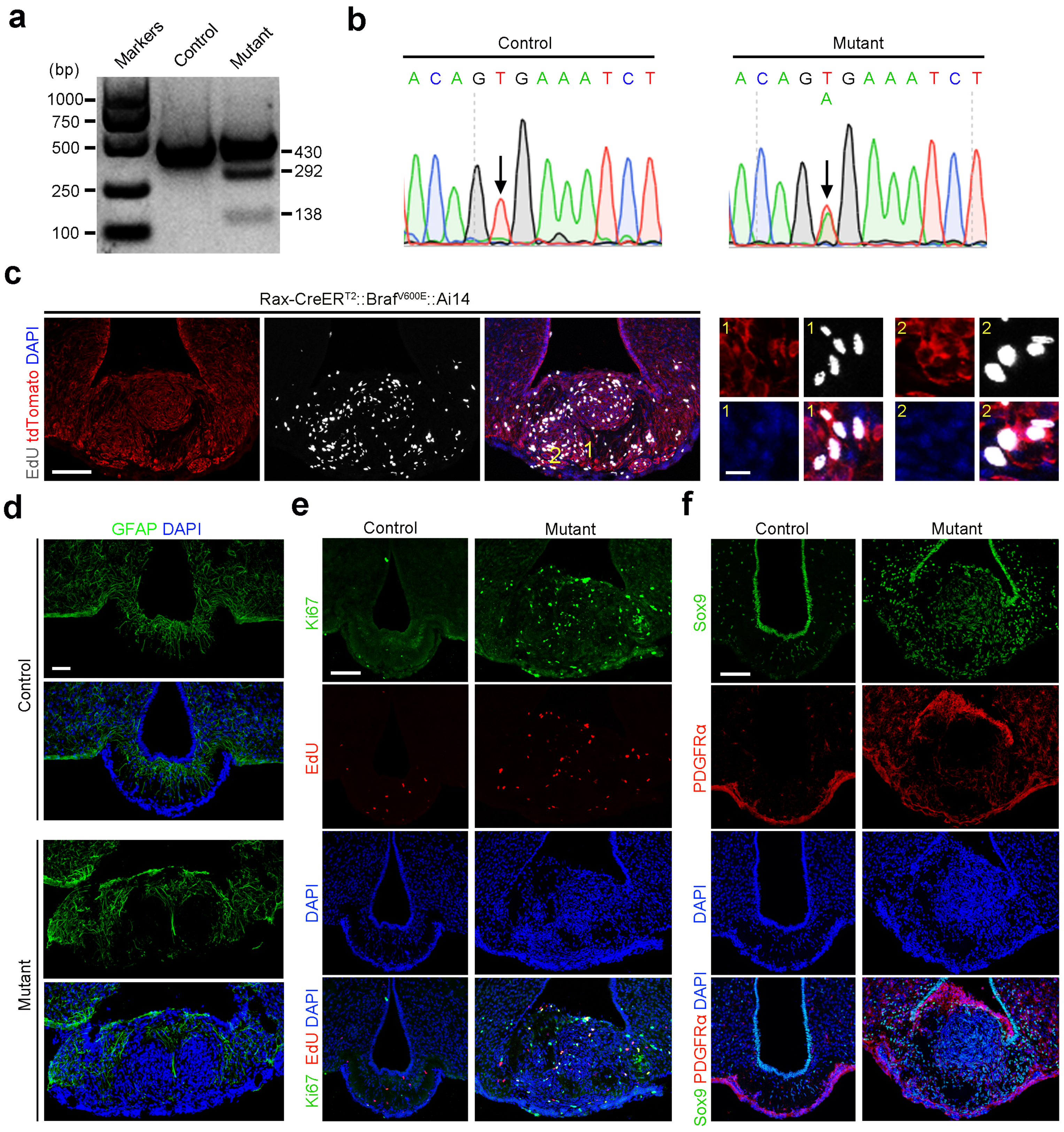

Supplementary Figure 7. Tanycyte-derived tumor does not display astrocytoma and oligodendrocytoma features. a, Gel electrophoresis of PCR amplified products cut by XbaI restriction enzyme. We microdissected tumor tissues, isolated mRNA, performed reverse transcription to obtain cDNA and amplified the target fragment by PCR for restriction endonuclease reactions. **b,** Sanger sequencing of target DNA fragment showing the heterozygous double peaks at the targeted base pair, indicated by arrows, in the induced Rax-CreER^{T2}::Braf^{V600E} mice. **c**, Sample confocal images stained for EdU and tdTomato in ME from Rax-CreERT2::BrafV600E::Ai14 mouse model at 2 mpi. In the central zone of neoplastic tissues, magnified images show that EdU^{+} tumor cells were predominantly positive for tdTomato (Right). Scale bars, 100 µm (left) and 10 µm (right). **d**, Sample images showing the expression of astrocyte marker GFAP outside the center zone of tumor. Scale bar, 50 µm. **e**, Representative confocal images showing the mitotic division of cells in center and marginal zone of tumor tissues. Scale bar, 100 µm. **f**, Sample images stained for stem cell marker Sox9 and OPC marker PDGFRα in control ME and tumor tissues. Scale bar, 100 µm. Source data are provided as a Source Data file.

Supplementary Figure 8

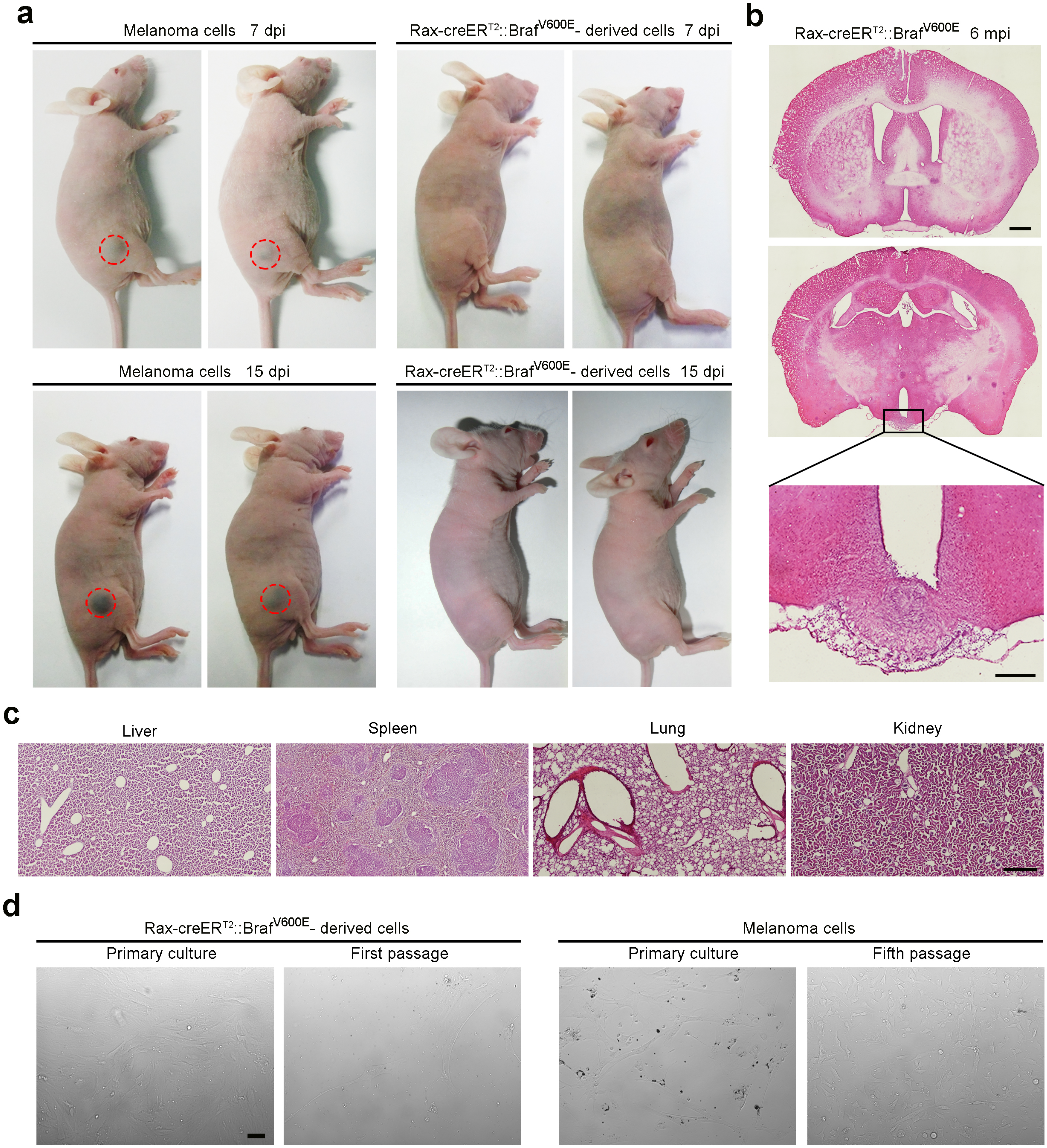

Supplementary Figure 8. Characterization of neoplastic properties in Rax-CreER^{T2}::Braf^{V600E} mice. **a**, Representative mice bearing xenograft tumors. Xenograft transplantation of tanycyte-derived tumor cells did not cause neoplasm in nude mice at 7 and 15 days post inoculation (dpi), but the transplantation of melanoma cells induced a significant subcutaneous neoplasm (highlighted by red dashed circles). **b**, Hematoxylin and eosin (HE) staining of brain sections from Rax-CreER^{T2}::Braf^{V600E} mutant mice at 6 mpi. Scale bars, 1 mm and 500 µm in magnified image. **c**, HE staining of liver, spleen, lung and kidney from Rax-CreER^{T2}::Braf^{V600E} mutant mice at 6 mpi. Scale bar, 200 µm. **d,** Sample optical imaging showing the morphology and density of cells arising from Rax-CreER^{T2}::Braf^{V600E} mutant mice (left) and melanoma animal models (right). Scale bar, 50 µm.

Supplementary Figure 9

a

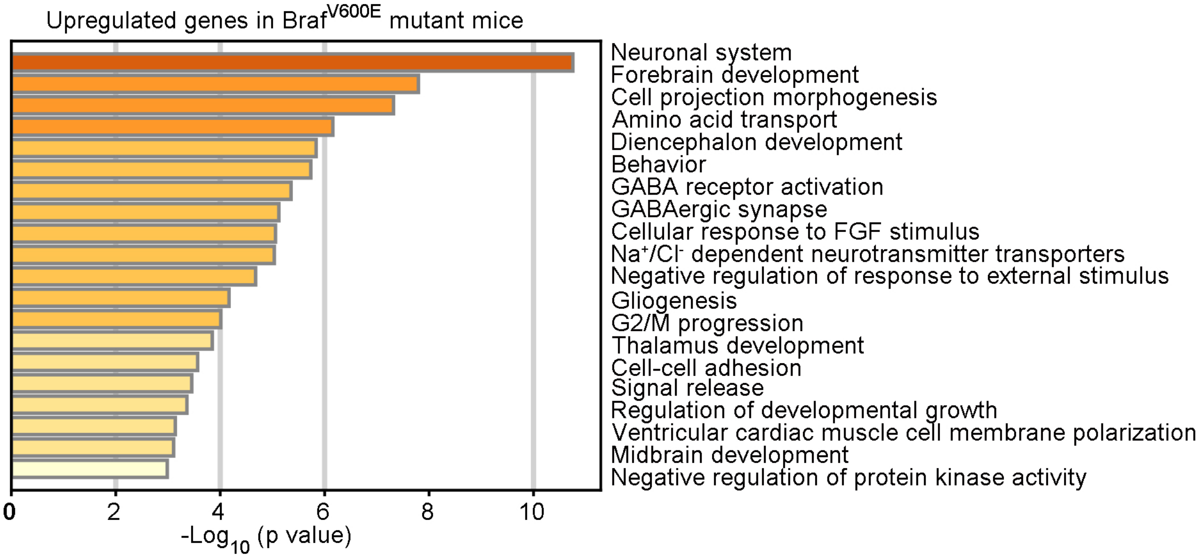

b

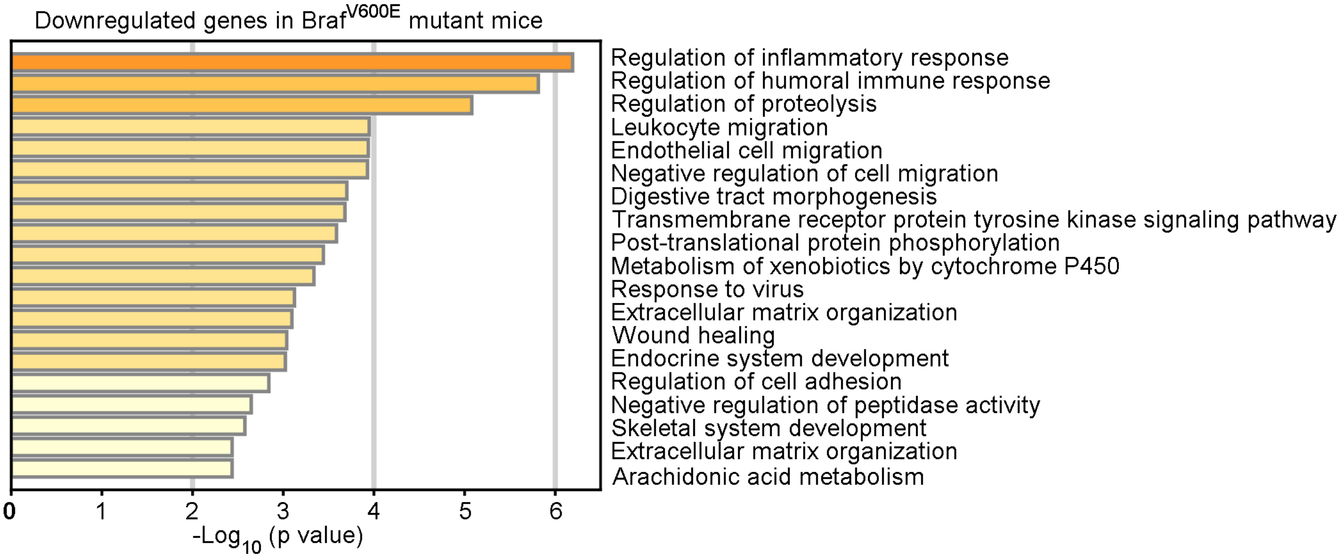

Supplementary Figure 9. Gene ontology analysis of dysregulated genes in mouse tumor tissues. Gene ontology analyses of upregulated (a) and downregulated (b) genes in the ME of Rax-CreER^{T2}::Braf^{V600E} mutant mice.

Supplementary Figure 10

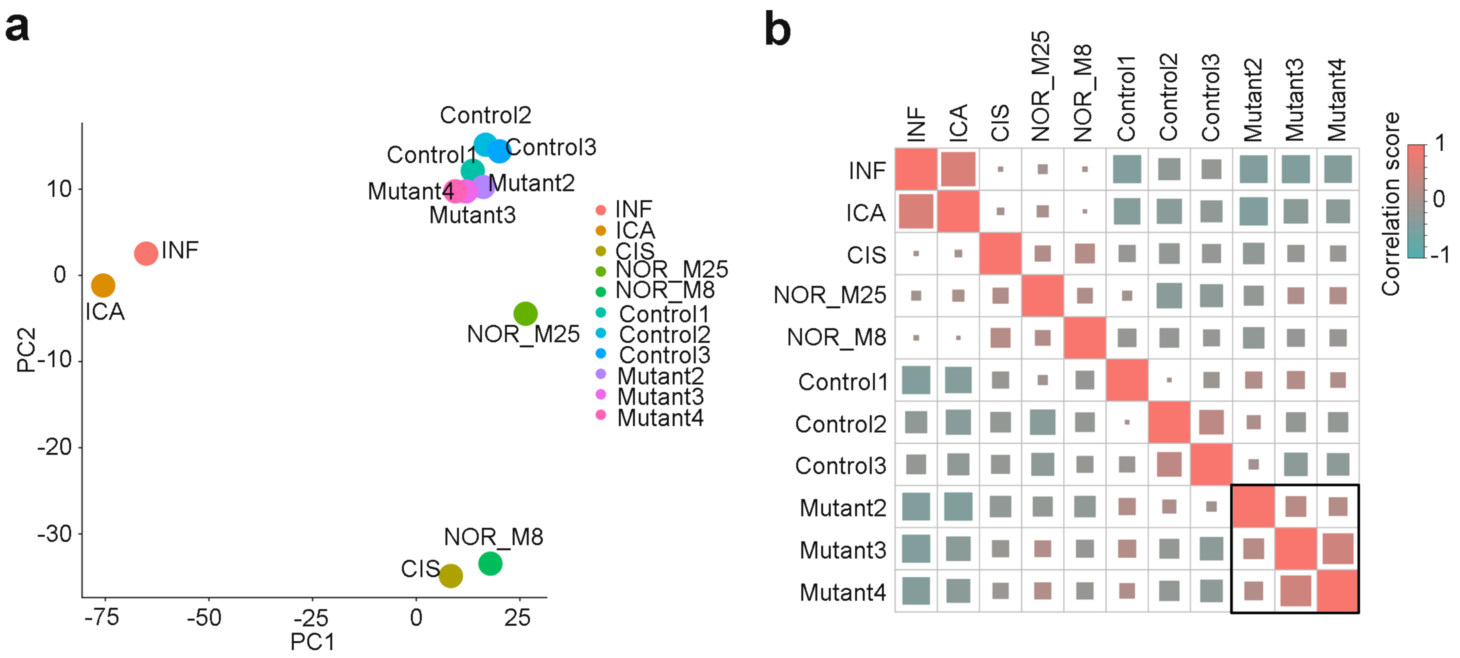

Supplementary Figure 10. Comparative transcriptomic analysis of tanycyte- and esophageal squamous cell-derived tumors. Principal component analysis (PCA) and Spearman's correlation analysis of bulk RNAseq data collected from normal tanycytes (Control group), tumors arising from tanycytes (Mutant group), normal esophageal squamous epithelia (NOR groups), inflammatory esophageal squamous epithelial cells (INF group), *in situ* esophageal squamous cell carcinoma (CIS group) and invasive esophageal squamous cell carcinoma (ICA group). The RNAseq data of NOR, INF, CIS and ICA groups were downloaded from a recently published study [2]. The bulk mRNA from normal cells, precancerous cells and tumor cells were collected, reverse transcribed and amplified with a similar procedure for next-generation sequencing. NOR, normal; INF, inflammation; CIS, carcinoma in situ; ICA, invasive carcinoma; M, month of mouse age.

Supplementary Data 1. Molecular signatures for different cell clusters.

Supplementary Data 2. Shared molecular markers among NSCs, ependymal cells and tanycytes.

Supplementary Data 3. Precise *p* values and sample size.

Supplementary Table 1. Primers used for genotyping and qPCR.

Primer IDs	Sequence (5' to 3')
Rax-CreER^{T2}-Common-F	CCCTGAGGCTAAACTTGCAG
Rax-CreER^{T2}-WT-R	AGGTGTCTAGGATGCCGTCT
Rax-CreER^{T2}-Mutant-R	AGGCAAATTTTGGTGTACGG
Igf1r$^{f/f}$ -F	CTTCCCAGCTTGCTACTCTAGG
Igf1r$^{f/f}$ -R	CAGGCTTGCAATGAGACATGGG
Braf^{V600E} -F	TGAGTATTTTTGTGGCAACTGC
Braf^{V600E} -R	CTCTGCTGGGAAAGCGGC
LSL-tdTomato (Ai14)-Mutant-F	CTGTTCCTGTACGGCATGG
LSL-tdTomato (Ai14)-Mutant-R	GGCATTAAAGCAGCGTATCC
iDTR-WT-F	AGTCGCTCTGAGTTGTTATCAG
iDTR-WT-R	TGAGCATGTCTTTAATCTACCTCGATG
iDTR-Mutant-F	TCATGTTTAGGTACCATAGGAGAGG
iDTR-Mutant-R	TGAACTTGTGGCCGTTTACG
GFAP-CreER^{T2}-Con-F	CTAGGCCACAGAATTGAAAGATCT
GFAP-CreER^{T2}-Con-R	GTAGGTGGAAATTCTAGCATCATCC
GFAP-CreER^{T2}-Tan-F	GCCAGTCTAGCCCACTCCTT
GFAP-CreER^{T2}-Tan-R	TCCCTGAACATGTCCATCAG
iDTA-Common-F	GTTATCAGTAAGGGAGCTGCAGTGG
iDTA-WT-R	GGCGGATCACAAGCAATAATAACC
iDTA-Mutant-R	AAGACCGCGAAGAGTTTGTCCTC
Braf target fragment V600E-F	GCTCGGCAGACTGCACAGGGCATGGATTAC
Braf target fragment V600E-R	TGAGGCACTCTGCCATTAATCTCTTCATGGC

Supplementary Table 2. Probes used for smFISH analysis

Primer IDs	Sequence (5' to 3')
Mia	GAGGAGGGCAGCAAACGGAAAGCCCTGCTAGGCCCTGAAA
Mia	GAGGAGGGCAGCAAACGGAATCGTCCGCACACAGCTTCCA

Mia	GAGGAGGGCAGCAAACGGAAGGAAATAGCCCAGGCGGGCT
Mia	GAGGAGGGCAGCAAACGGAAAAACAGACAAGACGACGATG
Mia	GAGGAGGGCAGCAAACGGAACCTCGTCCGCACACAGCTTC
Mia	GAGGAGGGCAGCAAACGGAAAGACATACACCACTTGGCCC
Mia	AGCTTGGGCATAGCTCGATCAAGAAGAGTCTTCCTTTACG
Mia	AGATAGGATGGCTGCATTCCAAGAAGAGTCTTCCTTTACG
Mia	CTCCCGGACAATGCTACTGGAAGAAGAGTCTTCCTTTACG
Mia	AGCCCTGCTAGGCCCTGAAAAAGAAGAGTCTTCCTTTACG
Mia	GGAGATAGGATGGCTGCATTAAGAAGAGTCTTCCTTTACG
Mia	ACGGCCCTTCAACTTGGAGAAAGAAGAGTCTTCCTTTACG
Mia	GAGGAGGGCAGCAAACGGAAAGCCCTGCTAGGCCCTGAAA

Supplementary References

1. Shah, P.T. et al. Single-Cell Transcriptomics and Fate Mapping of Ependymal Cells Reveals an Absence of Neural Stem Cell Function. Cell 173, 1045-1057 e1049 (2018).
2. Yao, J. et al. Single-cell transcriptomic analysis in a mouse model deciphers cell transition states in the multistep development of esophageal cancer. Nature communications 11, 3715 (2020).
