# Supplementary material for: Hypothalamic Rax+ tanycytes contribute to tissue repair and tumorigenesis upon oncogene activation in mice
Source: Nat Commun. 2021 Apr 16;12:2288. doi: 10.1038/s41467-021-22640-z (PMC8052410; doi:10.1038/s41467-021-22640-z)
Supplement: Supplementary file 3 — Description of Additional Supplementary Files [file 41467_2021_22640_MOESM3_ESM.pdf]

Description of Additional Supplementary Files

Supplementary Data 1. Molecular signatures for different cell clusters.

Supplementary Data 2. Shared molecular markers among NSCs, ependymal cells and tanycytes.

Supplementary Data 3. Precise *p* values and sample size.
